# Caught by Whole‐Exome Sequencing: Hemoglobin Sun Prairie in a Patient With Unexplained Hemolytic Anemia From Nepal

**DOI:** 10.1155/crh/9718800

**Published:** 2025-12-29

**Authors:** Prashun Upadhaya, Anjan Shrestha

**Affiliations:** ^1^ Department of Internal Medicine, Hemato-Oncology Unit, Maharajgunj Medical Campus, Institute of Medicine, Tribhuvan University Teaching Hospital, Kathmandu, Nepal, teachinghospital.org.np

**Keywords:** hemoglobinopathy, Hemoglobin Sun Prairie, hemolytic anemia, Nepal, unstable hemoglobin, whole-exome sequencing

## Abstract

**Background:**

Unstable hemoglobin leading to chronic hemolysis is a rare yet an important cause of hemolytic anemia and can be easily missed if not thought of during evaluation. Hemoglobin Sun Prairie is an extremely rare α‐globin mutation associated with hemolytic anemia.

**Case:**

We report a 22‐year‐old female from Nepal who presented with easy fatigability and jaundice since her childhood with worsening of symptoms for 2 months. Examination revealed pallor, icterus, and splenomegaly. Investigation showed anemia with low mean cell volume (MCV), reticulocytosis, indirect hyperbilirubinemia, and raised lactate dehydrogenase (LDH) level. Workup for etiology of hemolysis, including autoimmune, nutritional, RBC enzyme deficiency, and osmotic fragility, was negative. Hemoglobin electrophoresis was inconclusive. Whole‐exome sequencing identified a pathogenic mutation in HBA2 gene, confirming Hemoglobin Sun Prairie. To our knowledge, this represents the first reported case of Hemoglobin Sun Prairie from Nepal.

**Conclusion:**

This case highlights the importance of genetic testing in an unexplained hemolytic anemia. Genetic testing such as whole‐exome sequencing would help in the early identification of rare causes of hemolytic anemia which can guide in the genetic counseling and prevent unnecessary investigations and interventions.

## 1. Introduction

Hemoglobinopathies are the most common monogenic disease worldwide, caused by structural abnormalities or quantitative defects in the globin chains of hemoglobin, and include the common structural disorders such as sickle cell disease and the thalassemias [1]. The clinical spectrum of hemoglobinopathies ranges from an asymptomatic carrier state to life‐threatening anemia. In a clinically suspected case, diagnostic evaluations include complete blood count (CBC) with peripheral blood smear that show clues such as microcytic anemia, normal to high RBC count and RDW, microcytosis, target cells, anisopoikilocytosis, sickle cells, basophilic stippling, and nucleated RBCs [2]. High‐performance liquid chromatography (HPLC) or capillary electrophoresis (CE) helps to characterize the hemoglobin, whereas targeted mutation analysis or sequencing identifies the specific globin gene mutation [3]. Targeted laboratory series, hospital registries, and regional field surveys in Nepal show that thalassemias are unevenly distributed across the country, with higher frequencies among some ethnic groups and southern plains, and pose a significant public health burden [4–6].

## 2. Case Presentation

A 22‐year‐old female from eastern Nepal presented to our hematology clinic with complaints of generalized fatigue and intermittent yellowish discoloration of eyes since childhood which had increased for 2 months. She had been transfused with 3 units of packed red blood cell at the age of 12 years for anemia. There was no history of joint pain, rashes, photosensitivity, dark‐colored urine, or gall stone disease. She was born of a nonconsanguineous marriage, and her family history was unremarkable with no known cases of anemia, jaundice, blood disorders, or transfusion requirements.

On examination, the patient was pale and icteric with splenomegaly of 4 cm below the left subcostal margin. There was no lymphadenopathy or hepatomegaly. Cardiovascular and respiratory system examinations were otherwise normal.

Initial laboratory workup showed a hemoglobin level of 5.8 g/dL and an absolute reticulocyte count of 352,000/cu mm, with normal WBC and platelet counts. Peripheral blood smear revealed anisopoikilocytosis with polychromasia, tear‐drop cells, target cells, and few fragmented cells. Serum biochemistry revealed elevated LDH (694 U/L) and indirect hyperbilirubinemia (bilirubin total 110 μmol/L and bilirubin direct 17 μmol/L), with a normal iron profile. Initial investigations are shown in Table 1. A review of available prior records and serial laboratory results showed values similar to that at the presentation.

**Table 1:** Laboratory test parameters at presentation.

Hemoglobin	5.8 g/dL
WBC	5000/cu mm
Platelet	242,000/cu mm
Mean corpuscular volume (MCV)	76 fL
RBC count	2.57 million/cu mm
Absolute reticulocyte count	352,000/cu mm
Peripheral blood smear	Anisopoikilocytosis, microcytic hypochromic RBCs with polychromasia, tear‐drop cells, target cells, and few fragmented RBCs
Serum LDH	694 U/L
Bilirubin total	110 μmol/L
Bilirubin direct	17 μmol/L
Serum iron	110 μg/dL
Serum total iron binding capacity (TIBC)	203 μg/dL
Serum ferritin	70.2 ng/mL
Antineutrophilic antibody (ANA)	Negative
Direct Coombs test	Negative
Glucose‐6‐phosphate dehydrogenase (G6PD) level	18.66 U/g

**Figure 1:**
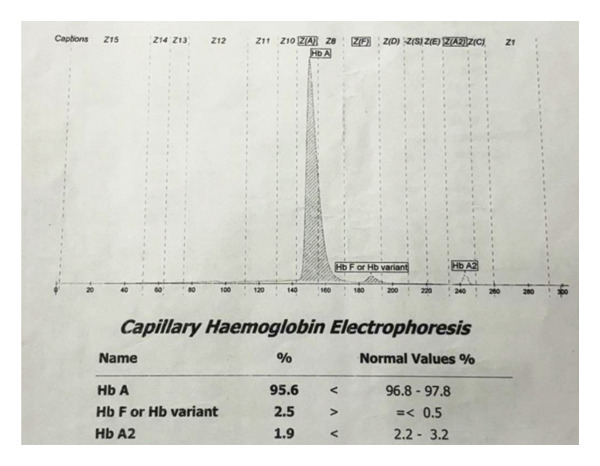
Capillary hemoglobin electrophoresis showing HbA 95.6%, Hb variant in the HbF/variant window 2.5%, and HbA2 1.9%.

The direct Coombs test was negative, and the G6PD level was normal. Capillary hemoglobin electrophoresis demonstrated a predominant HbA fraction of 95.6% (reference range: 96.8%–97.8%), a small additional fraction of 2.5% migrating in the HbF/variant window (reference range ≤ 0.5%), and an HbA2 fraction of 1.9% (reference range: 2.2%–3.2%) (Figure 1). The presence of a minor abnormal peak in the HbF/variant zone, in the context of ongoing hemolytic anemia and absence of β‐thalassemic indices, was suggestive of an unstable hemoglobin variant. To further delineate the etiology, whole‐exome sequencing (WES) was performed externally using the Illumina NovaSeq 6000 platform and analyzed with the DRAGEN pipeline for alignment (GRCh38), variant calling, and annotation, which identified a homozygous c.391G > C (p.Ala131Pro) mutation in the *HBA2* gene, consistent with Hb Sun Prairie, a rare α‐globin chain variant classified as “likely pathogenic” (Table 2). Additionally, a heterozygous c.2555C > T (p.Thr852Met) variant in the *SPTB* gene was detected and classified as a variant of uncertain significance. The combined findings confirmed the diagnosis of Hb Sun Prairie–associated hemolytic anemia. Family members were not available for genetic testing to confirm segregation of the mutation.

**Table 2:** Whole‐exome sequencing showing HBA2 mutation (Hb Sun Prairie) and additional SPTB variant^∗^.

Gene and transcript	Location	Variant	Zygosity/inheritance	OMIM phenotype	Clinical significance
HBA2 (+) NM_000517.6	Exon 3	c.391G > C (p.Ala131Pro)	Homozygous/autosomal recessive	Hb Sun Prairie	Likely pathogenic (PM2, PS4, PS3, PP5)
SPTB (−) NM_001355436.2	Exon 14	c.2555C > T (p.Thr852Met)	Heterozygous/autosomal dominant/autosomal recessive	Anemia, neonatal hemolytic, fatal, or near‐fatal/Elliptocytosis 3/Spherocytosis, Type 2	Uncertain significance^#^ (PM2)

^∗^Genetic test results are reported based on the recommendations of the American College of Medical Genetics.

^#^Due to the lack of clinical evidence for this variant, it is classified as a variant of uncertain significance.

## 3. Discussion

Hemoglobin Sun Prairie is a type of alpha chain variant that results due to G ⟶ C mutation in Codon 130 of α2 gene resulting in replacement of alanine residue by a proline residue in the H helix. The resulting α chain is unstable and precipitates within the red cell, causing hemolysis. Other manifestations include reticulocytosis, indirect hyperbilirubinemia, and splenomegaly all of which were present in our patient [7]. It has a recessive pattern of inheritance with heterozygous individual demonstrating asymptomatic thalassemia carrier phenotype, while homozygous state leads to chronic hemolytic anemia [8]. After its first description in a young patient of Indian origin with severe hemolytic anemia, only a handful of patients have been further reported worldwide [7, 9–11].

Conventional techniques such as HPLC or hemoglobin electrophoresis often fail to detect this variant, posing a diagnostic challenge [7]. Hence, molecular techniques, such as DNA sequencing, are often required. In resource‐limited settings like ours, diagnosis of hemoglobin variants relies on hemoglobin electrophoresis and HPLC. However, these methods may fail to clearly distinguish rare variants, especially when the abnormal peak is small or overlaps with HbF or HbA2. In Hb Sun Prairie, homozygous state of the pathogenic mutation leads to an abnormal peak and low HbA2 percentage [10]. In our case, CE demonstrated a small peak at the HbF region (2.5%) and a slightly reduced HbA2 level (1.9%), which prompted further evaluation. WES confirmed the pathogenic *HBA2* mutation consistent with Hb Sun Prairie, highlighting the importance of molecular diagnostics in suspected rare hemoglobinopathies.

Interestingly, our patient also harbored a heterozygous *SPTB* gene variant (c.2555C > T; p.Thr852Met), which is associated with hereditary spherocytosis and elliptocytosis, though its clinical significance in this case remains uncertain. While dual genetic findings can potentially contribute to phenotype severity, further functional studies are required to establish a causal relationship.

Unfortunately, family studies could not be performed, which would have strengthened the causal association of the identified variant. Though the patient is of Nepali origin, information on detailed ancestry and extended heritage could not be ascertained. Given Nepal’s proximity to Northern India where several hemoglobinopathy variants are prevalent, heritage data would have strengthened deeper epidemiologic interpretation.

To our knowledge, this is the first documented case of Hb Sun Prairie from Nepal, expanding the geographical spectrum of this rare hemoglobinopathy. Reporting such cases is crucial for raising awareness, guiding genetic counseling, and strengthening the role of advanced molecular testing in hematology practice.

## 4. Conclusion

We report the first case of Hb Sun Prairie–associated hemolytic anemia from Nepal, confirmed by hemoglobin electrophoresis and WES. This case highlights the importance of considering rare hemoglobin variants in the differential diagnosis of unexplained hemolytic anemia and underscores the role of molecular diagnostics in establishing a definitive diagnosis. Family studies, though not available in this instance, would further support genotype–phenotype correlation. Early recognition of such variants is essential for appropriate patient management and genetic counseling.

## Ethics Statement

This case report does not need any ethical approval.

## Consent

Written informed consent was obtained from the patient for publication of this case report.

## Disclosure

The authors have no affiliations with or involvement in any organization or entity with any financial interests in the subject matter or materials discussed in the manuscript.

## Conflicts of Interest

The authors declare no conflicts of interest.

## Author Contributions

Prashun Upadhaya: conceptualization, data curation, and writing–original draft.

Anjan Shrestha: review and editing.

## Funding

No funding was received for this manuscript.

## Data Availability

All relevant data supporting the findings of this case report are included within the article.
